# Violation of prophylactic vancomycin administration timing is a potential risk factor for rate of surgical site infections in cardiac surgery patients: a prospective cohort study

**DOI:** 10.1186/s12872-017-0506-5

**Published:** 2017-03-08

**Authors:** Paolo Cotogni, Cristina Barbero, Roberto Passera, Lucina Fossati, Giorgio Olivero, Mauro Rinaldi

**Affiliations:** 1 Department of Anesthesia and Intensive Care, S. Giovanni Battista Hospital, University of Turin, Via Giovanni Giolitti 9, 10123 Turin, Italy; 2 Department of Cardiovascular Surgery, S. Giovanni Battista Hospital, University of Turin, Turin, Italy; 3 Nuclear Medicine Unit, S. Giovanni Battista Hospital, University of Turin, Turin, Italy; 4 Microbiology and Virology Laboratory, S. Giovanni Battista Hospital, University of Turin, Turin, Italy; 5 Department of Surgical Sciences, S. Giovanni Battista Hospital, University of Turin, Turin, Italy

**Keywords:** Antimicrobial prophylaxis, Surgical wound infection, Postoperative infectious complications

## Abstract

**Background:**

Intensivists and cardiothoracic surgeons are commonly worried about surgical site infections (SSIs) due to increasing length of stay (LOS), costs and mortality. The antimicrobial prophylaxis is one of the most important tools in the prevention of SSIs. The objective of this study was to investigate the relationship between the timing of antimicrobial prophylaxis administration and the rate of SSIs.

**Methods:**

A prospective cohort study was carried out over 1-year period in all consecutive adult patients undergoing elective cardiac surgery. The population was stratified in patients whose antimicrobial prophylaxis administration violated or not the vancomycin timing protocol (i.e., when the first skin incision was performed before the end of vancomycin infusion). To compare SSI rates, the cohort was further stratified in patients at low and high risk of developing SSIs.

**Results:**

Over the study period, 1020 consecutive adult patients underwent cardiac surgery and according to study inclusion criteria, 741 patients were prospectively enrolled. A total of 60 SSIs were identified for an overall infection rate of 8.1%. Vancomycin prophylaxis timing protocol was violated in 305 (41%) out of 741 enrolled patients. SSIs were observed in 3% of patients without violation of the antimicrobial prophylaxis protocol (13/436) compared with 15.4% of patients with a violation of the timing protocol (47/305) (*P* < 0.0001). Patients at low risk with protocol violation had a higher occurrence of SSIs (*P* = 0.004) and mortality (*P* = 0.03) versus patients at low risk without protocol violation. Similarly, patients at high risk with protocol violation had a higher occurrence of SSIs (*P* < 0.001) and mortality (*P* < 0.001) versus patients at high risk without protocol violation. The logistic regression analysis showed that internal mammary artery use (*P* = 0.025), surgical time (*P* < 0.001), intensive care unit (ICU) LOS (*P* = 0.002), high risk of developing SSIs (*P* < 0.001) and protocol violation (*P* < 0.001) were risk factors for SSI occurrence as well as age (*P* = 0.003), logistic EuroSCORE (*P* < 0.001), ICU LOS (*P* < 0.001), mechanical ventilation time (*P* < 0.001) and protocol violation (*P* < 0.001) were risk factors for mortality.

**Conclusions:**

This study showed that violation of the timing of prophylactic vancomycin administration significantly increased the probability of SSIs and mortality from infectious cause in cardiac surgery patients.

## Background

The incidence of surgical site infections (SSIs) after cardiac surgery ranges differently according to the type of wound infection; specifically, superficial wound infection occurs in 2 to 20% of patients and deep sternal wound infection occurs in 0.25 to 5% [1–6].

Risk factors that have been linked to SSIs include features in the host such as advanced age, the presence of liver or lung dysfunction, cancer, diabetes mellitus and over- or undernutrition [7, 8]. Similarly, several operation characteristics can influence the risk of infection in cardiac surgery: skin antisepsis; length of operation; surgical technique; coronary artery bypass graft (CABG) surgery involving the use of a saphenous vein autograft that can carry bacteria from the harvest site deep into the cardiac operative site; use of the internal mammary artery (IMA) that deprives the sternum of blood supply; the use of prosthetic intracardiac or aortic implants; cardiopulmonary bypass or systemic cooling for myocardial protection; and invasive devices remaining after surgery (chest drains, pacing wires and intravenous catheters) [1, 8, 9]. Recent reports focused on an increasing number of infections caused by resistant Gram-positive pathogens, such as methicillin-resistant Staphylococcus aureus (MRSA) and coagulase-negative staphylococcus [4, 5, 10]. Compared with methicillin-sensitive Staphylococcus aureus mediastinitis, MRSA mediastinitis has up to an 11-fold increased mortality rate [5].

In patients undergoing cardiac surgery, an SSI is associated with increased morbidity, prolonged length of stay and increased costs with an in-hospital mortality rate of 10–20% [1, 6, 11]. Thus, many preventive measures were suggested as effective for reducing the incidence of SSIs, such as preoperative screening for carriage of multiresistant organisms (e.g., MRSA), antimicrobial prophylaxis, preoperative skin preparation, accurate surgical technique, postoperative glycemic control and wound management [9, 12]. Antimicrobial intravenous prophylaxis is routinely administered to patients undergoing cardiac surgery because the benefits of preoperative antibiotic administration in these patients have been clearly demonstrated in placebo-controlled studies [13]. However, the debate over choice, dose, duration and timing of antimicrobial prophylaxis protocol is still all the rage [14].

Seminal literature demonstrated that antimicrobial prophylaxis administered too late or too early reduced the efficacy of the antibiotic and may increase the risk of SSI [7]. The definite timing of administration of the first antibiotic dose has not been assessed in randomized controlled trials; however, there is a strong rationale supporting the need for the timely administration of preoperative antimicrobial prophylaxis [7, 11, 15]. Indeed, the timing of the administration of the prophylactic antibiotic is still an important issue for the cardiac surgical community [16–19], because despite the existence of published guidelines and locally agreed protocols for the antimicrobial prophylaxis administration, often there is a gap between what is recommended and what is practiced [15, 20].

The primary objective of this study was to investigate the relationship between the timing of antimicrobial prophylaxis administration —with respect to surgical incision time— and the rate of SSIs, comparing cardiac surgery patients at low and high risk of infection. This objective was related to a specific exploratory mandate received from our Hospital Infection Control Committee to evaluate our policy of antimicrobial prophylaxis in cardiac surgery.

## Methods

### Study design

A single-centre prospective cohort study was carried out in the Department of Cardiovascular Surgery of a 1200-bed tertiary care university hospital (S. Giovanni Battista Hospital). Over 1-year period, all consecutive adult patients undergoing cardiac surgery were assessed for eligibility. The exclusion criteria were renal dysfunction (on dialysis or creatinine clearance ≤30 mL/min, estimated by the Cockcroft-Gault formula); infectious diseases that required antibiotic therapy in the previous 2 weeks; heart and lung transplant surgery; solid or hematologic tumours as well as chemotherapy or radiation therapy in the previous 6 months; preoperative stay in intensive care unit (ICU) more than 24 h; allergy to cefazolin or vancomycin; and emergency operations.

The study protocol was reviewed and approved by our Institutional Ethics Committee (No. 0078553) and patients provided written informed consent before their enrolment. The work was conducted in compliance with Institutional Review Board/Human Subjects Research Committee requirements.

Our protocol of antimicrobial prophylaxis was a single 1000 mg cefazolin dose, diluted in 20 mL 0.9% NaCl solution, initiated 30 to 60 min before surgery and administered as a slow intravenous bolus; plus a single 1000 mg vancomycin dose, diluted in 100 mL 0.9% NaCl solution, started within 2 h before surgery and administered over 60 min intravenously infusion to prevent the release of histamine. A further three doses of cefazolin 1000 mg at 8-h intervals were given postoperatively, while no further vancomycin doses were administrated postoperatively. Since 2005, our protocol provides the choice to combine cefazolin with vancomycin for antimicrobial prophylaxis in patients undergoing cardiac surgery. The rationale for using vancomycin was an increased prevalence of MRSA infections, which exceeded 60% hospital-wide and isolates identified in cardiac surgery patients with SSIs. Antimicrobial prophylaxis is started in the preoperative holding area. Vancomycin protocol and timing of administration were chosen based upon recommendations of our Hospital Infection Control Committee according to the Sanford Guide [21] and the Society of Thoracic Surgeons Guidelines [16].

The study population was stratified in patients whose antimicrobial prophylaxis administration violated or not our vancomycin timing protocol. Antimicrobial prophylaxis timing protocol was considered as violated when the first surgical skin incision was performed before the end of the vancomycin infusion. A healthcare provider (i.e., physician, nurse or cardiovascular technician) was required to document the exact time the antibiotic infusion was started, as well anaesthesiologists or cardiac surgeons who recorded the exact time the first skin incision.

To compare SSI rates adequately, the cohort was further stratified in patients at low and high risk of developing SSIs according to the literature [1, 8, 10]. Specifically, patients were included in the high risk group in case of: (i) chronic liver disease (classified as Child-Pugh class B and C); (ii) insulin-dependent diabetes; (iii) body mass index (BMI) <17 or >40 kg/m^2^; (iv) steroid or other immunosuppressive drug use; (v) chronic obstructive pulmonary disease; and (vi) extracardiac arteriopathy (i.e., claudication, carotid occlusion or >50% stenosis, amputation for arterial disease and previous or planned intervention on the abdominal aorta, limb arteries or carotids). Thus, cardiac surgery patients were assigned to four groups according to SSI risk factors and violation of the timing of antimicrobial prophylaxis protocol administration as follows: (i) low risk patients without protocol violation; (ii) low risk patients with protocol violation; (iii) high risk patients without protocol violation; and (iv) high risk patients with protocol violation.

According to Centers for Disease Control and Prevention (CDC) guidelines [8], the definition of an SSI requires that one of the following criteria be met: (i) superficial (infection above the sternum with no bone involvement); (ii) deep (infection involving the sternum and organ/space such as mediastinitis); and (iii) leg donor site infections. Patients with SSI must have positive culture results of surgical sites or drainage from the mediastinal area or evidence of infection during surgical re-exploration or fever, sternal instability and positive blood culture results [8]. Other infectious complications were defined as bloodstream infection (BSI), lower respiratory tract infection (LRTI) and urinary tract infection (UTI) according to CDC guidelines. Perioperative management and skin preparation were standardized in our Department according to CDC guidelines. According to the literature, patients were followed up for 30 days after the surgical procedure [18, 19]. Mortality was defined as death during hospitalization or within 30 days after surgery from infectious cause.

#### Statistical analysis

The patients’ characteristics were analysed by the Fisher’s exact test for categorical variables, while by the Mann–Whitney test for continuous ones; all results for the latter were expressed as the median (range). Two different independent series of univariate/multivariate binary logistic regression models were used to estimate the odds of SSI occurrence and mortality within 30 days after surgery (dependent variables), evaluating as their risk factors: gender, IMA use, high risk of developing SSIs and protocol violation (independent categorical variables) as well as age, BMI, surgical time, logistic EuroSCORE, mechanical ventilation time and ICU length of stay (LOS) (independent continuous variables). All reported P values were obtained by the two-sided exact method, at the conventional 5% significance level. Data were analysed by R 3.3.2 (R Foundation for Statistical Computing, Vienna-A, http://www.R-project.org).

## Results

**Fig. 1:**
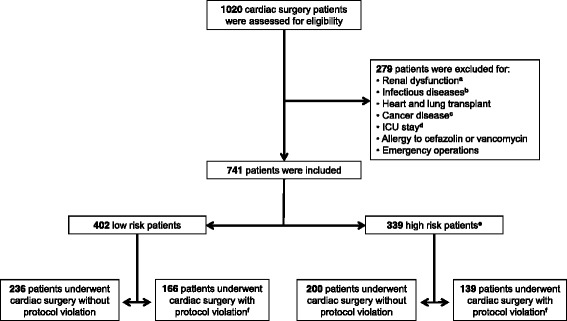
Study design. ^a^Dialysis or creatinine clearance ≤30 mL/min. ^b^Infectious diseases that required antibiotic therapy in the previous 2 weeks. ^c^Patients with solid or hematologic tumours, as well as patients underwent chemotherapy or radiation therapy in the previous 6 months. ^d^Preoperative stay in the intensive care unit (ICU) for more than 24 h. ^e^Patients were considered at high risk of developing surgical site infections in case of: chronic liver disease (classified as Child-Pugh class B and C); insulin-dependent diabetes; body mass index <17 or >40 kg/m^2^; steroid or other immunosuppressive drug use; chronic obstructive pulmonary disease; and extracardiac arteriopathy (i.e., claudication, carotid occlusion or >50% stenosis, amputation for arterial disease and previous or planned intervention on the abdominal aorta, limb arteries or carotids). ^f^Violation of antimicrobial prophylaxis timing protocol

Over the study period, 1020 consecutive adult patients underwent cardiac surgery. According to study inclusion criteria, 741 patients were prospectively enrolled, while 279 patients were excluded (Fig. 1). Main patients’ characteristics are reported in Table 1. According to variables considered as risk factors for infectious complications, 402 patients were considered at low risk of developing SSIs and 339 were considered at high risk. Of the 741 patients included in the study, antimicrobial prophylaxis timing protocol was violated in 305 patients (41.2%); specifically, in these patients the skin incision was performed before the end of the vancomycin infusion. No patients had vancomycin infusion more than 120 min prior to skin incision. No violation regarding cefazolin administration was observed.

**Table 1:** Patients’ characteristics

	Low risk (*n* = 402)			High risk (*n* = 339)		
	Without protocol violation	With protocol violation	*P*	Without protocol violation	With protocol violation	*P*
n	236	166		200	139	
Age, median (range)	70 (25–86)	70 (34–84)	0.51	71 (37–88)	71 (44–88)	0.50
Male gender, n (%)	149 (63)	100 (60.2)	0.63	124 (62)	74 (53.2)	0.13
BMI, kg/m^2^, median (range)	26 (18–40)	26 (18–39)	0.81	28 (17–43)	28 (17–41)	0.84
Diabetes, n (%)	46 (19)^a^	27 (16.3)^a^	0.41	50 (25)^b^	45 (32.4)^b^	0.17
COPD, n (%)	0	0	─	24 (12)	13 (9.3)	0.55
Hypertension, n (%)	151 (64)	108 (65.1)	0.91	132 (66)	99 (71.2)	0.37
Smoke, n (%)	28 (12)	30 (18.1)	0.11	52 (26)	48 (34.5)	0.12
Surgical time, min, median (range)	249 (119–593)	255 (132–495)	0.49	247 (140–442)	243 (152–430)	0.60
Surgical procedure, n (%)			0.08			0.11
CABG	72 (30.5)	58 (34.9)		70 (35)	53 (38.1)	
Valve	113 (47.9)	59 (35.5)		52 (26)	24 (17.3)	
CABG + Valve	34 (14.4)	30 (18.1)		24 (12)	12 (8.6)	
Other^c^	17 (7.2)	19 (11.5)		54 (27)	50 (36)	
Off-pump CABG, n (%)	21 (8.9)	8 (4.8)	0.17	11 (5.5)	9 (6.5)	0.89
Left IMA, n (%)	53 (22.4)	40 (24.1)	0.79	44 (22)	33 (23.7)	0.81
Both IMA, n (%)	17 (7.2)	8 (4.8)	0.44	13 (6.5)	9 (6.5)	>0.99
EuroSCORE additive, median (range)	5 (1–6)	5 (1–6)	0.80	8 (1–16)	8 (1–14)	0.72
EuroSCORE logistic, median (range)	4.8 (1–7.74)	4.6 (1–7.21)	0.41	9.7 (1–44.45)	9.9 (1–61.86)	0.37
Mechanical ventilation, h, median (range)	7 (2–912)	8 (6–415)	0.31	9 (7–816)	9 (8–711)	0.49
ICU stay, d, median (range)	1 (1–24)	1 (1–38)	0.52	1 (1–33)	1 (1–45)	0.30
RBC transfusions, n, median (range)	2 (0–9)	2 (0–6)	0.71	3 (0–11)	2 (0–10)	0.61

*BMI* body mass index, *COPD* chronic obstructive pulmonary disease, *CABG* coronary artery bypass grafting, *IMA* internal mammary artery, *EuroSCORE* European System for Cardiac Operative Risk Evaluation, *h* hours, *ICU* intensive care unit, *d* days, *RBC* red blood cell

^a^Non-insulin-dependent diabetes

^b^Insulin-dependent diabetes

^c^Aortic, atrial or ventricular septal defect repair, and congenital surgery

Table 2 shows infectious complications. SSIs were 8.1%: two-thirds were superficial wound infections of the chest, deep infections were 25% and few were at a donor site. SSIs were observed in 3% of patients without violation of the antimicrobial prophylaxis protocol (13/436) compared with 15.4% of patients with a violation of the timing protocol (47/305) (*P* < 0.0001). Patients at low risk with protocol violation had a higher occurrence of SSIs (*P* = 0.004), BSIs (*P* = 0.01) and mortality (*P* = 0.03) versus patients at low risk without protocol violation. Patients at high risk with protocol violation had a higher occurrence of SSIs (*P* < 0.001), BSIs (*P* < 0.001), LRTIs (*P* < 0.001), UTIs (*P* < 0.001) and mortality (*P* < 0.001) versus patients at high risk without protocol violation. Patients at high risk without violation of the antimicrobial prophylaxis protocol had a higher occurrence of SSIs (*P* = 0.0 4), BSIs (*P* = 0.04), LRTIs (*P* < 0.001) and mortality (*P* = 0.04) versus patients at low risk without protocol violation. Patients at high risk with protocol violation had a higher occurrence of SSIs (*P* < 0.001), BSIs (*P* < 0.001), LRTIs (*P* < 0.001), UTIs (*P* < 0.001) and mortality (*P* = 0.003) versus patients at low risk with protocol violation.

**Table 2:** Infectious complications

	Low risk (*n* = 402)		High risk (*n* = 339)		
	Without protocol violation	With protocol violation	Without protocol violation	With protocol violation	Total
N	236	166	200	139	741
SSI, n (%)	3 (1.3)	12 (7.2)^a^	10 (5)^b^	35 (25.2)^c,d^	60 (8.1)
Superficial, n	1	8	4	25	38 (63.3)
Deep, n	2	2	5	6	15 (25)
Donor site, n	0	2	1	4	7 (11.7)
BSI, n (%)	2 (0.8)	8 (4.8)^e^	12 (6)^b^	49 (35.2)^c,d^	71 (9.6)
LRTI, n (%)	7 (3)	8 (4.8)	25 (12.5)^f^	39 (23.1)^c,d^	79 (10.7)
UTI, n (%)	0	1 (0.6)	3 (1.5)	15 (10.8)^c,d^	19 (2.6)
Mortality^§^, n (%)	3 (1.3)	8 (4.8)^g^	9 (4.5)^b^	20 (14.4)^c,h^	40 (5.4)

*SSI* surgical site infection, *BSI* bloodstream infection, *LRTI* lower respiratory trait infection, *UTI* urinary trait infection

^§^During hospitalization or within 30 days after surgery from infectious cause

^a^ *P* = 0.004 versus low risk group without protocol violation

^b^ *P* = 0.04 versus low risk group without protocol violation

^c^ *P* < 0.001 versus high risk group without protocol violation

^d^ *P* < 0.001 versus low risk group with protocol violation

^e^ *P* = 0.01 versus low risk group without protocol violation

^f^ *P* < 0.001 versus low risk group without protocol violation

^g^ *P* = 0.03 versus low risk group without protocol violation

^h^ *P* = 0.003 versus low risk group with protocol violation

The logistic regression analysis showed that IMA use (*P* = 0.025), surgical time (*P* < 0.001), ICU LOS (*P* = 0.002), high risk of developing SSIs (*P* < 0.001) and protocol violation (*P* < 0.001) were risk factors for SSI occurrence (Table 3) as well as age (*P* = 0.003), logistic EuroSCORE (*P* < 0.001), ICU LOS (*P* < 0.001), mechanical ventilation time (*P* < 0.001) and protocol violation (*P* < 0.001) were risk factors for mortality (Table 4).

**Table 3:** Risk factors for surgical site infections (SSIs)

	Logistic regression Univariate models			Logistic regression Multivariate model		
	Odds ratio	95% CI	*P*	Odds ratio	95% CI	*P*
Body mass index	1.07	1.02-1.13	0.007	1.01	0.94-1.08	0.781
Internal mammary artery use	1.85	1.08-3.17	0.026	2.11	1.10-4.04	0.025
Surgical time	1.01	1.01-1.02	<0.001	1.01	1.01-1.02	<0.001
Intensive care unit LOS	1.12	1.09-1.16	<0.001	1.06	1.02-1.10	0.002
High risk^a^	3.95	2.16-7.22	<0.001	4.70	2.32-9.53	<0.001
Protocol violation^b^	5.93	3.15-11.17	<0.001	7.03	3.41-14.52	<0.001

*CI* Confidence interval, *LOS* length of stay

^a^High risk of developing SSIs according to the literature [1, 8, 10]; in case of: (i) chronic liver disease (classified as Child-Pugh class B and C); (ii) insulin-dependent diabetes; (iii) body mass index <17 or >40 kg/m2; (iv) steroid or other immunosuppressive drug use; (v) chronic obstructive pulmonary disease; and (vi) extracardiac arteriopathy (i.e.,claudication, carotid occlusion or >50% stenosis, amputation for arterial disease and previous or planned intervention on the abdominal aorta, limb arteries or carotids)

^b^Antimicrobial prophylaxis timing protocol was considered as violated when the first surgical skin incision was performed before the end of the vancomycin infusion

**Table 4:** Risk factors for mortality^a^

	Logistic regression Univariate models			Logistic regression Multivariate model		
	Odds ratio	95% CI	*P*	Odds ratio	95% CI	*P*
Age	1.13	1.07-1.19	<0.001	1.15	1.05-1.26	0.003
EuroSCORE logistic	1.24	1.17-1.31	<0.001	1.21	1.11-1.33	<0.001
Intensive care unit LOS	1.25	1.20-1.31	<0.001	1.14	1.07-1.21	<0.001
Mechanical ventilation time	1.34	1.24-1.45	<0.001	1.18	1.08-1.29	<0.001
Protocol violation^b^	3.57	1.79-7.14	<0.001	10.16	2.48-41.58	<0.001

*CI* Confidence interval, *LOS* length of stay

^a^During hospitalization or within 30 days after surgery from infectious cause

^b^Antimicrobial prophylaxis timing protocol was considered as violated when the first surgical skin incision was performed before the end of the vancomycin infusion

Ninety-two pathogens isolated in 60 SSIs are shown in Table 5. Specifically, Gram-positive, Gram-negative and fungi were isolated in 48, 40 and 12%, respectively. Pathogens isolated in SSIs by groups are depicted in Table 6. Methicillin-resistant Staphylococci (*aureus*, coagulase-negative and *hominis*) accounted for 28% of pathogens. When we studied methicillin-resistance or vancomycin susceptibility of Gram-positive isolates according to the timing of antimicrobial prophylaxis none of the differences between groups in the rate of such resistances reached statistical significance. No clusters of any specific pathogen were noted during the study period.

**Table 5:** Pathogens isolated in 60 surgical site infections

Pathogens, n	92
Gram-positive organisms, n (%)	44 (48)
*Staphylococcus aureus*, n	23
Coagulase-negative staphylococci, n	11
*Enterococcus* spp, n	9
Streptococci, n	1
Gram-negative organisms, n (%)	37 (40)
Fungi, n (%)	11 (12)

**Table 6:** Pathogens isolated in surgical site infections by groups

	Low risk (*n* = 402)		High risk (*n* = 339)		Total
	Without protocol violation	With protocol violation	Without protocol violation	With protocol violation	
SSI, n (%)	3/236 (1.3)	12/166 (7.2)	10/200 (5)	35/139 (25.2)	60/741 (8.1)
Pathogen, n	3	24	12	53	92
Gram-positive, n (%)	1 (33)	11 (46)	5 (42)	27 (51)	44 (48)
Methicillin-sensitive, n	1	3	2	12	18
Methicillin-resistant, n	0	8	3	15	26
Vancomycin susceptibility					
MIC ≤1, n	1	9	3	17	30
MIC =2, n	0	2	1	9	12
MIC ≥4, n	0	0	1	1	2
Gram-negative, n (%)	1 (33)	12 (50)	4 (33)	20 (38)	37 (40)
Fungi, n (%)	1 (33)	1 (4)	3 (25)	6 (11)	11 (12)

Multiple pathogens were identified in some patients; therefore, total pathogens identified do not add up to the total number of SSIs. *SSI* Surgical Site Infection, *MIC* minimum inhibitory concentration

## Discussion

In this study, cardiac surgery population was divided in patients at high risk of developing infections because of well-known risk factors and patients at low risk. As expected, patients at high risk (i.e., patients with severe comorbidities, immunosuppressive therapy, severe obesity or malnutrition) had a significant higher occurrence of SSIs as well as of BSIs, LRTIs and mortality compared with patients at low risk, independently of violation of the antimicrobial prophylaxis protocol. This finding reflects a population of patients who were more severely ill and therefore at higher risk for postoperative infectious complications.

The Society of Thoracic Surgeons Practice Guidelines on antibiotic prophylaxis in cardiac surgery recommended that in the setting of the institutional presence of a ‘high incidence’ of MRSA, it would be reasonable to combine a β-lactam (cefazolin) with a glycopeptide (vancomycin) for prophylaxis (Class IIB recommendation, Level of Evidence C) [16]. Optimal dosage regimens of vancomycin and protocol of administration still remain controversial [16, 22]. The Society of Thoracic Surgeons Guidelines mentioned that any of the following doses and durations may be used: 1000 mg, 1500 mg, or 15 mg/kg; and 24 h versus 48 h or 1 dose versus 2 doses [16, 22]. Specifically, guidelines for appropriate dosing of prophylactic antibiotics stated that ‘In patients for whom vancomycin is an appropriate prophylactic antibiotic for cardiac surgery, a dose of 1 to 1.5 g or a weight-adjusted dose of 15 mg/kg administered intravenously slowly over 1 h, with completion within 1 h of the skin incision, is recommended’ (Class I, Level of Evidence A) [16]. Similarly, the 2011 American College of Cardiology/American Heart Association guideline for CABG surgery recommended that ‘Antibiotic prophylaxis should be initiated 30 to 60 min before surgery, usually at the time of anaesthetic induction, except for vancomycin, which should be started 2 h before surgery and infused slowly’ [6]. Finally, the Scottish Intercollegiate Guidelines Network, updated April 2014, stated that ‘Vancomycin should be given by intravenous infusion starting 90 min prior to skin incision’ (Class B recommendation) [9].

Several studies investigated the association between measure(s) applied for reducing the rate of SSIs and their occurrence [23, 24]. Our study focused on the timing of antimicrobial prophylaxis; specifically, on the relationship between the first skin incision and the end of vancomycin infusion. We found that the initial surgical incision was performed before the vancomycin infusion had been completed in nearly 40% of patients. Generally, the reason for the violation of the antimicrobial prophylaxis protocol was due to our policy to start antimicrobial prophylaxis in the preoperative holding area under supervision of anaesthesiologists. This policy was adopted in our Cardiovascular Surgery Unit following the occurrence of some relevant adverse drug reactions due to vancomycin administration (i.e., mainly hypotension; occasionally, red man syndrome) [23, 24].

Differently, in the Garey’s study [25] cardiac surgery patients were assigned to five groups on the basis of the relation between the start time of the vancomycin infusion and the time of the initial surgical incision. In this study, antibiotic prophylaxis was started in the preoperative holding area only for the first surgical case of the day and in admission unit for all subsequent cases immediately prior to transferring the patient to the preoperative holding area. These Authors reported that of the 2048 patients in the study, 0.7% received vancomycin 0–15 min before incision, 8.6% 16–60 min before incision, 43.4% 61–120 min before incision, 34.2% 121–180 min before incision and 13.1% >180 min before incision.

The relationship between the timing of antimicrobial prophylaxis and the occurrence of SSIs has been studied with conflicting results. The Surgical Care Improvement Project measure assesses compliance for antimicrobial prophylaxis administration initiated within 60 min (or 120 min for vancomycin) prior to surgical incision [11]. The choice of the preincision 60-min window for antimicrobial prophylaxis was based on two types of evidence: pharmacokinetics of the antibiotics and one large cohort study analyzing the association between timing of antibiotic administration and SSIs in several types of surgical procedures [11].

However, following studies investigating this relationship did not clearly demonstrate the superiority of the 60-min window [17–20]; in particular, some studies showed lower risk of SSI with shorter times between antibiotic administration and skin incision. Garey et al. reported that SSI developed in 26.7% of cardiac surgery patients who received vancomycin 0–15 min before incision, 3.4% of patients between 16 and 60 min before incision, 7.7% of patients between 61 and 120 min before incision, 6.9% of patients between 121 and 180 min before incision and 7.8% of patients >180 min before incision [25]. Steinberg et al. in an observational study (43.6% were cardiac patients) found a trend toward lower risk of SSI occurring when antimicrobial prophylaxis with vancomycin or cephalosporins were given within 60 and 30 min prior to incision, respectively [18]. Hawn et al. in a retrospective study in noncardiac surgery patients found that the SSI risk was not significantly associated with prophylactic antibiotic timing [19].

SSIs are still among the most severe complications in cardiac surgery patients. The overall SSI rate observed in our study was 8.1%, which was similar or lower to that previously reported [1–3]. The main finding of our study was that violation of the timing of vancomycin prophylaxis protocol was a significant risk factor for development of SSI in patients undergoing cardiac surgery. Specifically, when the first surgical skin incision was performed before the end of the vancomycin infusion, we observed a 5-fold increased rate of SSIs both in low and high risk patients.

Nosocomial infections occur in 10 to 20% of cardiac surgery patients [6]; however, while SSIs incur significant morbidity and costs but rarely lead to death, conversely, postoperative LRTI, BSI and endocarditis are more frequently correlated with mortality [15]. In our study, we also found a consistent relationship between violation of vancomycin prophylaxis timing protocol and rates of postoperative infectious complications as well as mortality from infectious cause. Specifically, BSIs and mortality were increased 6-fold and more than 3-fold, respectively both in low and high risk patients. Moreover, LRTIs and UTIs were increased 2-fold and 7-fold, respectively in high risk patients. Also for mortality, violation of the timing of vancomycin prophylaxis protocol was a significant risk factor.

Actually, before starting this study we did not suspect that the timing of vancomycin prophylaxis administration was being violated at this rate as well as that this violation was associated with a significantly increased rate of SSIs, postoperative infectious complications and mortality from infectious cause. However, the overall SSI rate and mortality observed in our study were similar or lower to those previously reported in other studies in cardiac surgery patients [1–6].

Policies and practices aimed at reducing the risk of SSIs include performing surveillance for SSIs as well as measuring and providing feedback to healthcare providers on the rates of compliance with process measures, including antimicrobial prophylaxis [7, 12]. This study let us to discover that the violation of the protocol was due to the start of antimicrobial prophylaxis in the preoperative holding area. Indeed, the information obtained in this study was reported to our healthcare providers and has altered practice patterns for avoiding the persistent risk of violation of prophylactic vancomycin administration timing.

### Strengths and limitations of the study

If compared with previous studies, our study has some relevant features: (1) it was a prospective study; (2) data were collected through a clinical study and not from a database or registry; (3) antibiotic timing data were collected in ‘real-time’ in the operating room and not from the patient chart; (4) only cardiac surgery patients were enrolled; (5) all patients received the same prophylactic administration of antibiotics; (6) the rate of BSIs, LRTIs, UTIs and mortality from infectious cause were also investigated; (7) it was concluded in only 12 months and (8) no patient was lost to follow-up. Moreover, to the best of our knowledge, this is the first study investigating the relationship between the rate of SSIs in cardiac surgery patients and the presence or absence of violation of the timing of antimicrobial prophylaxis administration, comparing patients at low and high risk of infections.

The study presented several limitations. First, it was a single-centre study. Second, neither a calculation was made on the number of subjects required nor an interim analysis was conducted since the study was designed by our statistician to be continuous over one year. Specifically, the duration of a year was necessary to enrol an adequate number of patients (i.e., 741) to obtain statistically significant differences among the groups. As a matter of fact, the earlier studies enrolled a number of patients ranging among 2048 and 4472 [18, 25].

Moreover, being an observational study, there was no randomization of patients to the two groups (with and without protocol violation), although the characteristics of patients in the two groups turned out not to be statistically different. Finally, in the study period patients were not screened for S. *aureus* colonization prior to surgery.

## Conclusions

The variability in antimicrobial prophylaxis timing significance among results reported in the literature suggests that the association between timing and SSIs is greatly related to the surgical population, the antibiotic(s) and the timing intervals investigated.

Despite some limitations, our study showed that violation of the timing of prophylactic vancomycin administration significantly increased the probability of SSIs and mortality from infectious cause in patients undergoing cardiac surgery.

## Abbreviations

BMI: Body mass index
BSI: Bloodstream infection
CABG: Coronary artery bypass graft surgery
CDC: Centers for Disease Control and Prevention
EuroSCORE: European system for cardiac operative risk evaluation
ICU: Intensive care unit
IMA: Internal mammary artery
LOS: Length of stay
LRTI: Lower respiratory tract infection
MRSA: Methicillin-resistant Staphylococcus aureus
SSI: Surgical site infection
UTI: Urinary tract infection
